# Case Report: Application of ^18^F-FDG PET/CT in identifying plasmacytoma in monoclonal gammopathy associated peripheral neuropathy

**DOI:** 10.3389/fnume.2024.1446780

**Published:** 2024-08-14

**Authors:** Jiequn Weng, Jie Lin, Chong Sun

**Affiliations:** ^1^ Department of Neurology, Yuyao People’s Hospital of Zhejiang Province, Yuyao, Zhejiang, China; ^2^ Department of Neurology, Huashan Hospital, Fudan University, Shanghai, China

**Keywords:** monoclonal gammopathy associated peripheral neuropathy, ^18^F-FDG PET/CT, multiple myeloma, monoclonal gammopathy of undetermined significance, chronic inflammatory demyelinating polyneuropathy

## Abstract

Peripheral neuropathy is a prevalent complication in plasma cell disorders, posing significant diagnostic and therapeutic challenges. This study presents three cases initially diagnosed with chronic inflammatory demyelinating polyneuropathy (CIDP). Despite initial symptom regression post-immunomodulatory treatment, the patients exhibited progressive neurological deficits. Advanced laboratory evaluation confirmed monoclonal protein presence, yet traditional diagnostic methods, including bone marrow biopsy and flow cytometry, yielded normal results. Utilizing ^18^F-FDG PET/CT, we identified multiple hypermetabolic vertebral lesions, which upon biopsy, confirmed the diagnosis of plasmacytoma. Our findings underscore the utility of PET/CT as a reliable diagnostic tool for monoclonal gammopathy associated neuropathy, advocating for its consideration in cases with equivocal diagnosis. When the diagnosis is in doubt, biopsy of a lesion may facilitate early and accurate diagnosis, potentially influencing treatment strategies and patient outcomes.

## Introduction

Peripheral neuropathy commonly accompanies plasma cell disorders, ranging from premalignant monoclonal gammopathy of undetermined significance (MGUS) to overt multiple myeloma (MM) (1). These disorders hallmark the secretion of monoclonal immunoglobulin (M protein). Neuropathy in these patients is heterogeneous, with approximately half resembling CIDP, posing significant diagnostic challenges (2). The over-reliance on electrodiagnostic studies and initial treatment response can be misleading. Chaudhry et al. proposed a diagnostic approach for monoclonal gammopathy associated peripheral neuropathy (3), but routine procedures often fail to identify the underlying pathophysiology. Meanwhile, ^18^F-FDG PET/CT was identified as an imaging technique with high reliability in terms of sensitivity and specificity for the detection of sites of clonal plasma cells. Here, we report three cases initially misdiagnosed as CIDP but ultimately diagnosed with MM through PET/CT and biopsy, emphasizing the potential role of PET/CT in diagnosing monoclonal gammopathy associated peripheral neuropathy.

## Case report

### Case 1

A 22-year-old man presented with progressive numbness and weakness in the feet for 2 years. He was diagnosed with CIDP and treated with intravenous methylprednisolone and obtained a regression of weakness by 30%. Later, motor and sensory symptoms progressed to the knees and the hands, despite treatment with intravenous immunoglobulin (IVIg) and plasmapheresis. Neurological examination showed distal weakness grade 2/5 in lower limbs and grade 4/5 in upper limbs, with generalized areflexia. Proprioception and vibration sense, as well as pinprick and temperature sensations, were absent distal to the knee.

**Figure 1:**
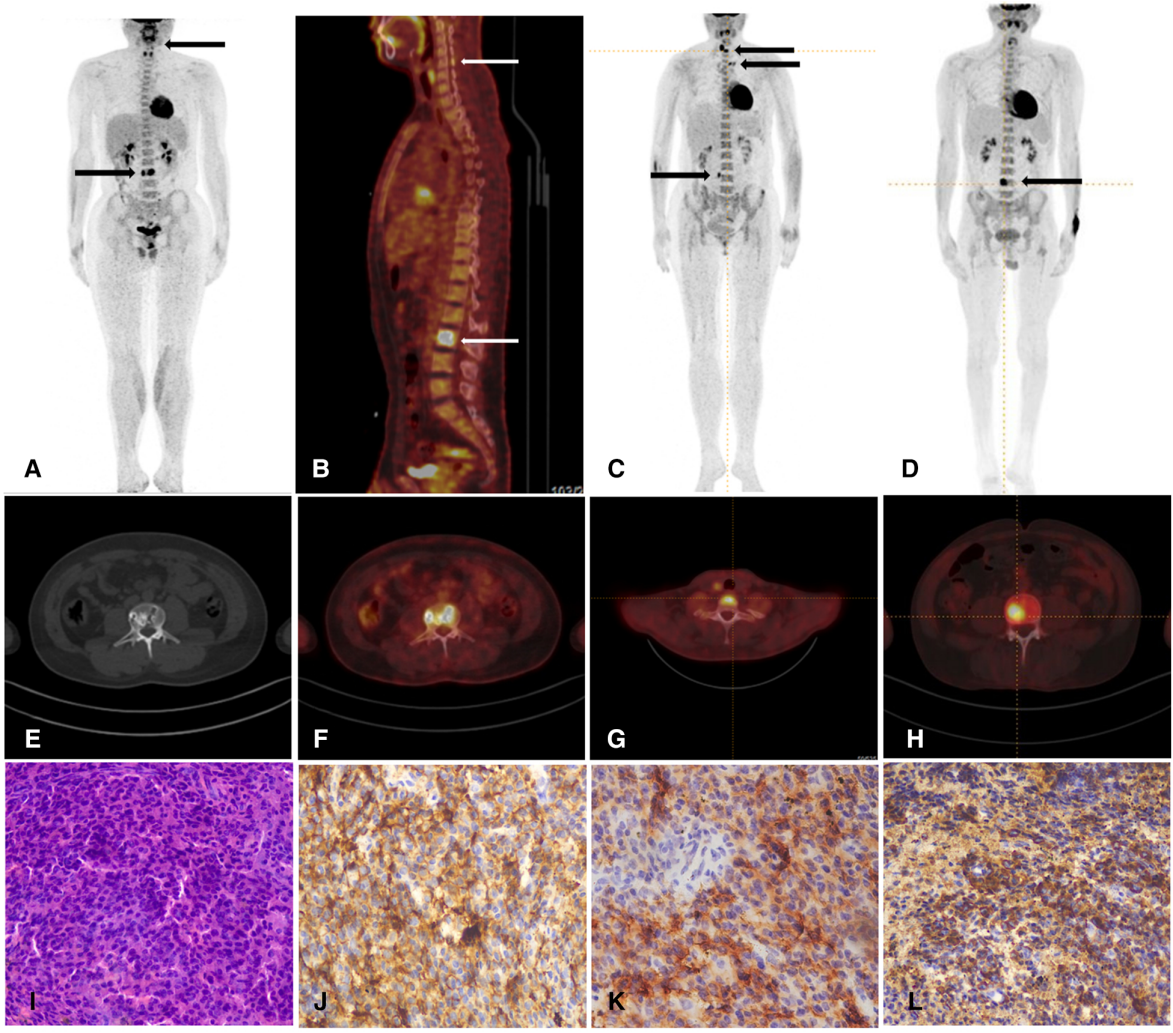
(**A**,**B**) Whole-body PET/CT shows high metabolism of 18F-FDG in the third lumbar vertebral body (SUV max 19.6) (arrows) and spinous process of the fifth cervical vertebra (SUV max of 4.5) in patient 1. (**C**) Whole-body PET/CT shows high metabolism of 18F-FDG in the seventh cervical vertebral body, the left transverse process of the third thoracic vertebra, and the right transverse process of the fourth lumbar vertebra (SUV max 8.5) (arrows) in the patient 2. (**D**) Whole-body PET/CT shows high metabolism of 18F-FDG in the fourth lumbar vertebral body in patient 3. (**E**,**F**) Axial PET/CT fused images demonstrated hypermetabolic lumber vertebral body lesions in patient 1. (**G**,**H**) Axial PET/CT fused images demonstrated hypermetabolic cervical vertebra in patient 2 and lumber vertebra in patient 3. (**I**) The histopathology of HE staining reveals lymphocyte hyperplasia in patient 1. These myeloma cells were filled with atypical blastic cells, containing a round nucleus. (**J**,**K**,**L**) The immunohistochemical results show positive staining of CD138, CD38, and λ (HE × 40) and negative staining of κ, CD3 and CD20.

Laboratory evaluation revealed normal complete blood count, hepatic and renal function, and blood glucose. Evidence for infections such as syphilis and HIV was not found. Vasculitic screens, anti-gangliosides and sulfatide (GS) antibodies, nodal and paranodal antibodies, and anti-MAG antibodies were negative. Cerebral spinal fluid protein was elevated (0.8 g/L). Serum immunofixation electrophoresis (IEP) identified an IgG λ monoclonal component. MRI of the brachial and lumbosacral plexus was normal. Nerve conduction studies showed slowing of motor and sensory conduction velocities and conduction blocks in median and ulnar nerves. Bone marrow biopsy from the iliac bone and flow cytometry were normal. ^18^F-FDG PET/CT showed intense uptake of mixed lytic and sclerotic lesions in the third lumbar and cervical vertebral bodies (Figure 1). A fluoroscopy-guided needle biopsy of the lumbar vertebral body confirmed plasma cell myeloma, with the accumulation of monoclonal plasma cells and the immunohistochemical staining [CD38 (+), CD138 (+), Lambda (+), Kappa (−), and CD20(−)] (Figure 1). Treatment with lenalidomide, ixazomib, and dexamethasone, followed by autologous hematopoietic stem cell transplantation (ASCT), resulted in moderate improvement in walking 6 months later.

### Case 2

A 60-year-old woman presented with 3 months of foot numbness followed by increasing difficulty in walking. She was initially diagnosed with CIDP and treated with IVIg and obtained 20% regression. She was admitted with progressive motor weakness and tingling in her limbs. Neurological examination revealed lower extremity weakness (3/5), absent proprioception and vibration sense distal to the knee, and generalized areflexia.

Laboratory studies revealed normal routine hematologic and blood chemistry parameters. CSF analysis showed increased protein (1,891 mg/L) and normal cell count. Serum IEP revealed a monoclonal IgG λ. Electrophysiologic nerve conduction studies disclosed diffuse decreased motor and sensory conduction velocities with increased amplitude. Percutaneous bone marrow was normal. ^18^F-FDG PET/CT showed intense uptake of mixed lytic and sclerotic bone lesions in the seventh cervical vertebral body, the left transverse process of the third thoracic vertebra, and the right transverse process of the fourth lumbar vertebra (Figure 1). Biopsy and histological examination of the vertebral lesion confirmed plasmacytoma and immunohistological stains showed IgG lambda light chains in the tumor cells. Chemotherapy with ixazomib and prednisone resulted in marked neurological improvement within 1-year follow up.

### Case 3

A 36-year-old man presented with 2 years of sensorimotor quadriparesis. Initial symptoms included paresthesias, dysesthesias, and distal lower extremity weakness, progressing to proximal lower extremities and distal upper extremities. He had moderate pain in the feet and ankles, worsening on movement. He was diagnosed as CIDP and treated with IVIg, without any improvement. Neurological examination revealed normal cranial nerves, significantly diminished distal muscle power (2-/5), and proximal muscle power (4/5), with generalized areflexia. Sensation was diminished in both upper and lower limbs in a stocking pattern.

Laboratory investigations revealed normal routine hematologic and blood chemistry profiles. CSF analysis showed increased protein (1,284 mg/l), and screening for endocrinopathies revealed hyperprolactinemia. Serum IEP identified a monoclonal IgG λ, with normal percutaneous bone marrow. Additional blood tests, including the Vascular Endothelial Growth Factor (VEGF), measured 912.27 pg/ml. Nerve conduction studies confirmed the presence of severe axonal demyelinating sensorimotor polyneuropathy. Given the lack of response to CIDP treatment and the presence of paraneoplastic indicators, an ^18^F-FDG PET/CT scan was performed, revealing intense uptake of a sclerotic lesion in the fourth lumbar vertebra (Figure 1). Biopsy and histological examination of the vertebral lesion led to the diagnosis of plasmacytoma. The patient received treatment with bortezomib and dexamethasone, followed by ASCT, which lead to a moderate improvement in approximately 1 year.

## Discussion

Our three patients initially presented with chronic motor sensory neuropathy, and were misdiagnosed as CIDP. The association between neuropathy and myeloma is well-established (4). However, when it is the sole initial symptom in many patients, underdiagnosis (i.e., failure to detect the low level of the M protein) or confusion with CIDP often occurs (3). The clinical “red flags” such as the presence of a M protein, and rapid symptom deterioration despite immunomodulatory treatment (5) should push the neurologist to evaluate for other causes of neuropathy (6, 7). In our cases, the identification of serum monoclonal IgG λ and extramedullary bone lesions via ^18^F-FDG PET/CT was pivotal for the correct diagnosis of MM. Establishing the causal or incidental relationship between monoclonal protein and peripheral neuropathy poses a challenge. An approach to the evaluation of a patient with an M protein identified in conjunction with peripheral neuropathy was suggested (3). Nevertheless, there is a lack of consensus regarding the role and timing of performing testing other than the routine procedures carried out at diagnosis of monoclonal gammopathy associated neuropathy. Given this heterogeneity and its potential to affect clinical care decisions, more comprehensive imaging is highly desirable.

With the combination of its metabolic properties with anatomic reliability, PET/CT provides high sensitivity and specificity to detect further diffuse and focal bone marrow infiltration (8). ^18^F-FDG PET/CT has become a standard technique in the diagnosis, staging, and management of MM and other clonal proliferative plasma cell disorders (9). Numerous studies have underscored its utility in the initial work-up for MM, highlighting a sensitivity and specificity in detecting bone marrow involvement and skeletal damage within the range of 80%–100% (10, 11). ^18^F-FDG PET/CT is indispensable for confirming a suspected diagnosis of solitary plasmacytoma, and differentiating between smouldering and active MM (12, 13). Additionally, it serves as an excellent prognostic tool for patients with newly diagnosed and relapsed or refractory MM, and is instrumental in evaluating and monitoring treatment responses due to its capacity to discern metabolically active from inactive sites of clonal proliferating plasma cells (14, 15).

**Figure 2:**
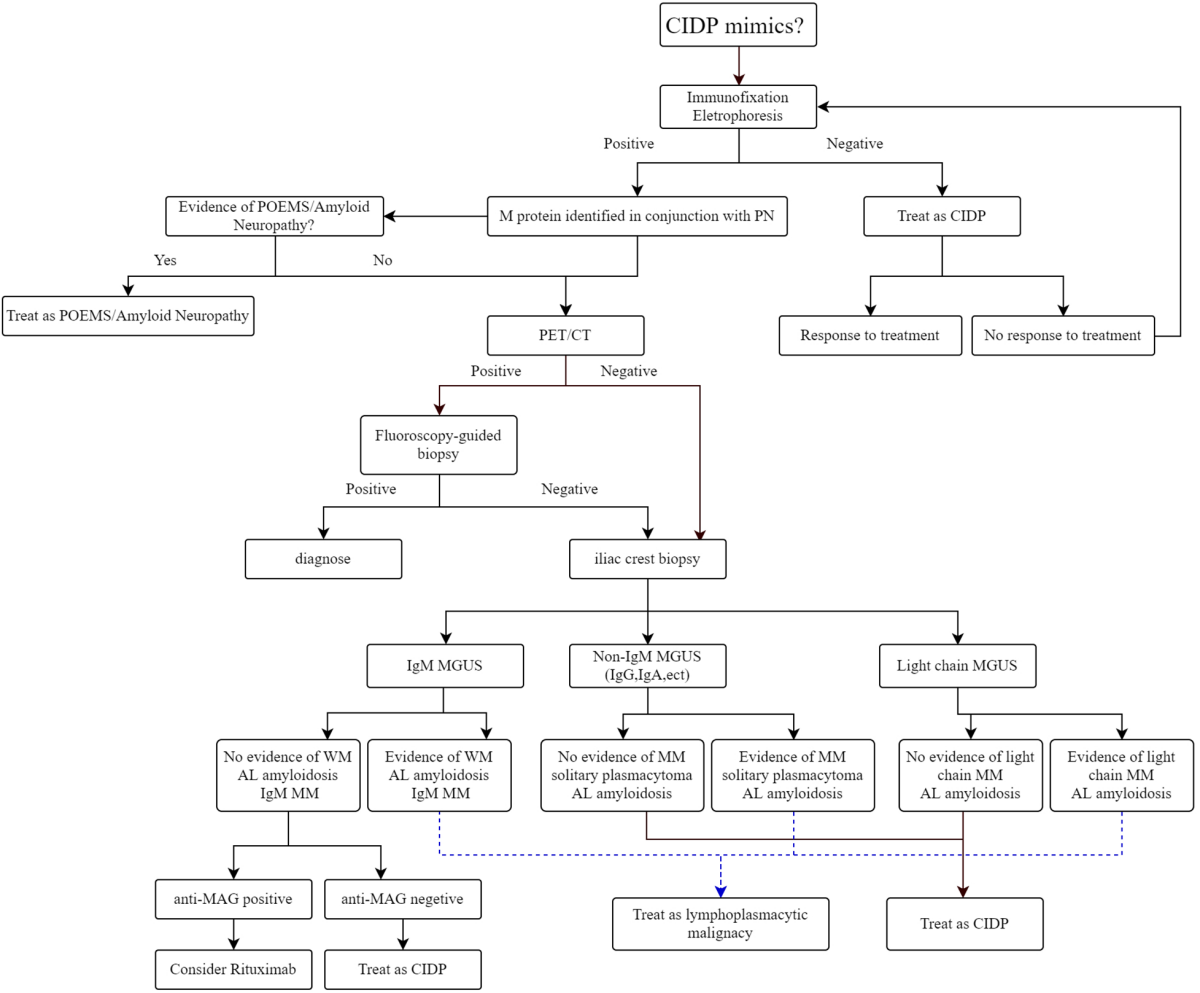
Approach to diagnosis and management of monoclonal gammopathy associated peripheral neuropathy.

Furthermore, whole-body imaging modalities have demonstrated that MM is not uniformly distributed within the bone marrow. In fact, studies indicate that approximately 60% of patients exhibit focal or patchy plasma cell accumulation and bone destruction (16). The incidence of extramedullary multiple cell mass arising from the underlying bones (e.g., ribs, vertebrate, skull, sternum, pelvis) in MM patients varies from 7% to 34% (17). Compared to systemic bone x-ray, PET/CT significantly increases the detection rate of these lesions (18). The International Myeloma Working Group (IMWG) recommends that whole-body imaging should be done in all patients with suspected MM and in MGUS patients with high risks, which include an M-protein of 1.5 g/dl or more and an abnormal free light chain ratio with non-IgM MGUS (19). Although the literature about the possible role of ^18^F-FDG PET/CT in patients with MGUS is scarce, one recent study noted its benefit in the early detection of various accompanying disorders in MGUS patients (20). On ^18^F-FDG PET/CT scans, the presence of focal or diffuse areas of abnormally increased radiopharmaceutical uptake in approximately 10% of MGUS patients. The rarity of MGUS in young patients (age <40 years at diagnosis), with a prevalence of less than 0.3%, suggests that M protein presence in this demographic may be linked to a heightened risk of malignancy (21). Considering these findings, and the possible connection of MGUS to an increased risk of developing hematological and solid malignancies, we propose a diagnostic approach for patients with monoclonal gammopathy associated neuropathy to be evaluated by PET/CT, especially for patients with young age (Figure 2). Despite the superiority of PET/CT, the relatively expensive diagnostic procedure, reduced availability, and relatively higher radiation exposure restrict its widespread use. The cost-effectiveness of incorporating PET/CT into the diagnostic protocol requires further investigation. Furthermore, a major limitation of ^18^F-FDG PET/CT is the absence of standardized imaging criteria. To address these challenges, an international consortium of European haematologists, nuclear medicine physicians, and physicists has initiated a standardization project for PET/CT scan interpretation criteria, which is based on the foundational IMPeTUs proposal (22).

## Conclusion

The accurate identification of underlying plasma cell disorders in patients with monoclonal gammopathy associated neuropathy is essential for appropriate management. Our case series indicates that ^18^F-FDG PET/CT is a potentially valuable diagnostic tool in the evaluation of these patients. A biopsy of a suspected lesion can aid early diagnosis and guide appropriate treatment.

## Data Availability

The original contributions presented in the study are included in the article/Supplementary Material, further inquiries can be directed to the corresponding author.
